# Supplementary material for: Expression of recombinant human glutamylating TTLLs in human cells leads to differential tubulin glutamylation patterns, with only TTLL6 disrupting microtubule dynamics
Source: PLoS One. 2026 Mar 2;21(3):e0339922. doi: 10.1371/journal.pone.0339922 (PMC12952574; doi:10.1371/journal.pone.0339922)
Supplement: S1 Fig — (DOCX) [file pone.0339922.s001.docx]

**Supporting Infortmation**

**Expression of recombinant human glutamylating TTLLs in human cells leads to differential tubulin glutamylation patterns, with only TTLL6 disrupting microtubule dynamics**

Mohamed Aghyad Al Kabbani^1, 2,^, Pragya Jatoo^3, *^, Anne-Kathrin Klebl^3^, Bert M. Klebl^3^, Hans Zempel^1, 2, *^

1 Institute of Human Genetics, Faculty of Medicine and University Hospital Cologne, University of Cologne, Cologne, Germany

2 Center for Molecular Medicine Cologne (CMMC), Faculty of Medicine and University Hospital Cologne, University of Cologne, Cologne, Germany

3 Lead Discovery Center GmbH, Otto-Hahn-Str. 15, 44227 Dortmund, Germany

* Correspondence to: Dr. Dr. Hans Zempel, [hans.zempel@uk-koeln.de](mailto:hans.zempel@uk-koeln.de)

* Correspondence to: Dr. Pragya Jatoo, [jatoo@lead-discovery.de](mailto:jatoo@lead-discovery.de)


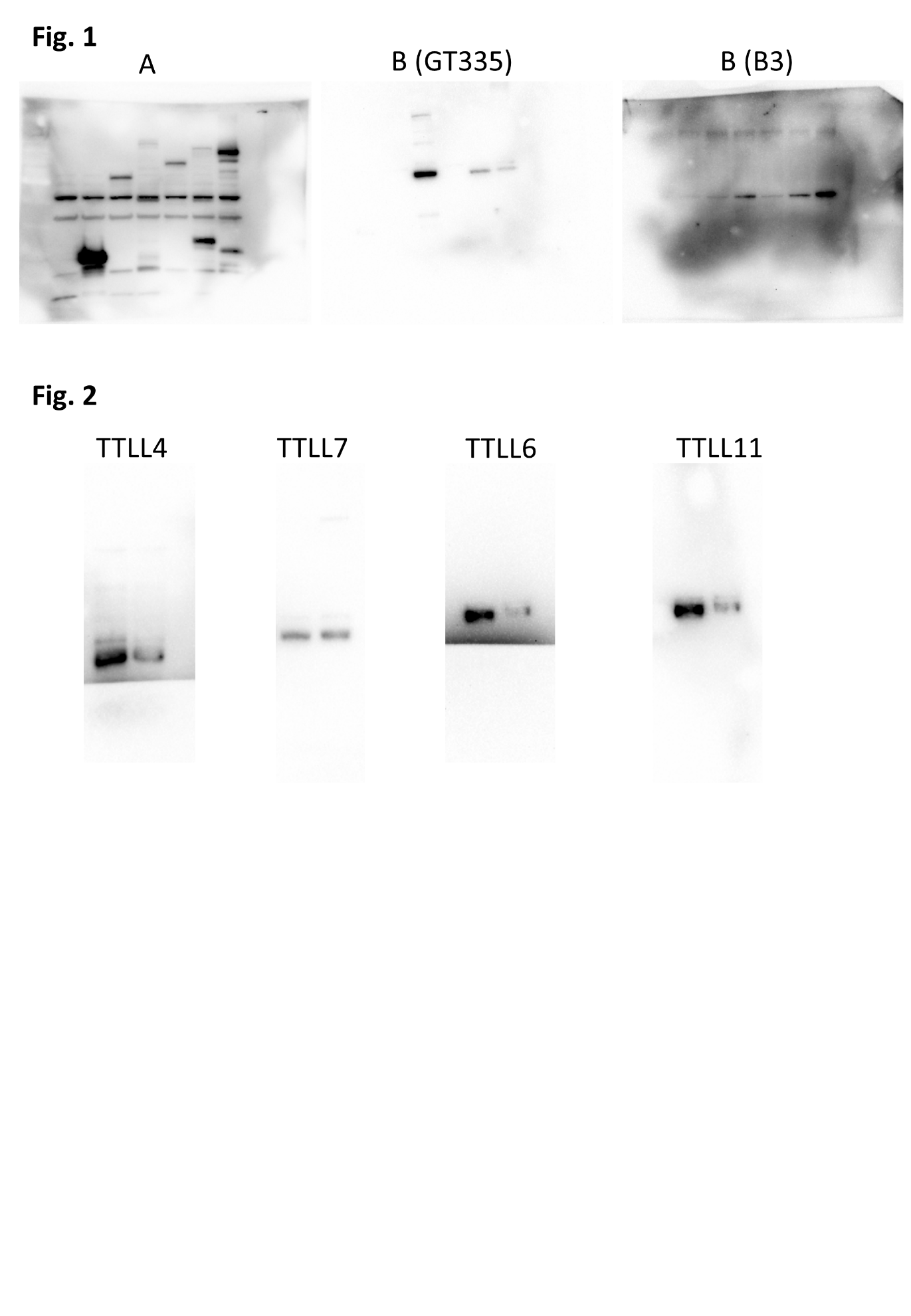


**Fig S1.** Raw uncropped blots from Fig 1 and 2.

All datasets included in this study are available in the corresponding Excel file “**Supporting Information - Raw Datasets**”.
